# Cognitive Impairment, Dementia and Depression in Older Adults

**DOI:** 10.3390/jcm15031198

**Published:** 2026-02-03

**Authors:** Yoo Jin Jang, June Ho Chang, Daa Un Moon, Hong Jin Jeon

**Affiliations:** 1 Department of Psychiatry, Samsung Medical Center, Sungkyunkwan University School of Medicine, 81 Irwon-ro, Gangnam-gu, Seoul 06351, Republic of Korea; yjjjs519@naver.com (Y.J.J.); jhchang36@gmail.com (J.H.C.); 2 Department of Psychiatry and Neurosciences, Charité Campus Mitte, Charité—Universitätsmedizin Berlin, 10117 Berlin, Germany; daaunmoon@googlemail.com; 3 Department of Consultation-Liaison Psychiatry and Psychosomatic Medicine, University Hospital Zurich, University of Zurich, 8091 Zurich, Switzerland; 4 Department of Health Sciences and Technology, Medical Device Management and Research, Clinical Research Design and Evaluation, Samsung Advanced Institute for Health Sciences & Technology, Sungkyunkwan University School of Medicine, 81 Irwon-ro, Gangnam-gu, Seoul 06351, Republic of Korea

**Keywords:** late-life depression, mild cognitive impairment, dementia, behavioral and psychological symptoms of dementia, cognitive decline

## Abstract

This narrative review integrates longitudinal cohort studies, neuroimaging and biomarker research, and major clinical trials to examine how depression and cognitive decline interact across the dementia continuum. Depression and cognitive impairment frequently co-occur in late life and exhibit substantial clinical and biological overlap. Meta-analytic and large population-based cohort studies consistently show that late-life depression increases the risk of mild cognitive impairment and dementia, with stronger associations observed for vascular dementia than for Alzheimer’s disease. Neurobiological studies implicate cerebrovascular pathology, neuroinflammation, hypothalamic–pituitary–adrenal axis dysregulation, and fronto-subcortical circuit dysfunction as key mechanisms linking depressive symptoms to later cognitive decline. In a subset of older adults, new-onset depression—particularly when accompanied by executive dysfunction, subjective cognitive decline, or high white-matter hyperintensity burden—are associated with an increased likelihood of near-term cognitive decline and dementia, although evidence for a definitive prodromal state remains limited. Depression is also highly prevalent as part of the behavioral and psychological symptoms of dementia, occurring in 30–50% of individuals with Alzheimer’s disease and even higher proportions in dementia with Lewy bodies or frontotemporal dementia. Comorbid depression in dementia accelerates cognitive and functional decline, increases neuropsychiatric burden, and worsens quality of life for patients and caregivers. Therapeutically, antidepressant treatment may confer modest benefits on mood and selected cognitive domains (e.g., processing speed and executive function) in non-demented older adults, whereas in established dementia, antidepressant efficacy is limited. In contrast, cholinesterase inhibitors, memantine, and multimodal non-pharmacological interventions yield small but measurable improvements in depressive or apathy-related symptoms. Emerging disease-modifying therapies for Alzheimer’s disease have demonstrated cognitive benefits, but current trial data provide insufficient evidence regarding effects on depressive symptoms, highlighting an important gap for future research. These findings underscore the need for stage-specific, integrative strategies to address the intertwined trajectories of mood and cognition in aging.

## 1. Introduction

Cognitive impairment and depression are among the most prevalent neuropsychiatric conditions in late life. They frequently co-occur and exert reciprocal influences on each other [1,2]. Epidemiological studies have consistently shown that late-life depression is associated with a higher risk of developing mild cognitive impairment (MCI) and dementia [3], and with accelerated trajectories of cognitive decline once impairment has emerged [4], while cognitive decline often contributes to the emergence or persistence of depressive symptoms [5]. Both conditions share overlapping clinical features—such as deficits in attention, executive function, and processing speed—that are implicated across neuropsychological, neuroimaging, and longitudinal studies and are therefore clinically salient for differential diagnosis [6,7,8]. The concept of “pseudodementia”, once used to describe reversible cognitive impairment secondary to depression, has evolved to recognize that depressive symptoms may reflect an early manifestation of dementia in some cases, highlighting the blurred boundary between affective and neurodegenerative disorders in older adults [9].

Neurobiologically, depression and dementia exhibit several shared pathophysiological pathways—most consistently cerebrovascular burden and dysregulation of the hypothalamic–pituitary–adrenal (HPA) axis, with additional contributions from neuroinflammation and reduced neuroplasticity—although the strength of evidence varies across imaging, biomarker, and longitudinal cohort studies [10,11,12,13]. Late-onset depression, commonly defined as first-onset depressive episodes occurring after approximately 60–65 years of age [2], has been linked to frontostriatal and limbic system alterations as well as white matter hyperintensity (WMH) burden, suggesting a “vascular depression” subtype that bridges mood disturbance and cognitive decline [14,15]. Conversely, in established dementia, depressive symptoms are often part of the behavioral and psychological symptoms of dementia (BPSD), reflecting both reactive responses to functional loss and neurobiologically driven changes associated with the spread of pathology into emotion-regulating networks, with relative contributions varying across dementia subtypes [16,17,18]. These bidirectional and stage-dependent interactions underscore the need for an integrative framework that considers depression and cognitive decline as part of a continuum rather than distinct entities.

Understanding the interplay between mood and cognition has important therapeutic implications. Accumulating evidence suggests that antidepressant treatment may improve not only mood but also specific domains of cognitive performance, particularly executive function and processing speed, in non-demented older adults [19,20]. Cognitive enhancers are associated with small improvements in behavioral and psychological symptoms of dementia such as agitation and apathy, while emerging disease-modifying antibody therapies remain of translational interest but currently lack evidence for meaningful mood-related outcomes [21,22]. This narrative review aims to synthesize current evidence on the shared and distinct cognitive profiles of depression and dementia, explore the neurobiological and clinical features of depression as a prodromal or comorbid manifestation of dementia, and discuss the bidirectional treatment effects that bridge mood and cognition in late life. Beyond summarizing existing evidence, this review aims to provide an integrative conceptual framework that differentiates depression as a risk factor, prodromal manifestation, or component of BPSD across disease stages, and to map clinical phenotypes to underlying biology and treatment implications. The review is based on a focused evaluation emphasizing late-life onset, longitudinal evidence, biomarker-informed studies, and clinically actionable markers from epidemiological research, neuroimaging and biomarker studies, and major clinical trials relevant to depression and dementia in older adults.

## 2. Literature Identification, Scope of Review, and Terminology

This narrative review was informed by a focused literature search of PubMed, and Google Scholar covering publications from January 2000 through March 2025. Key search terms included combinations of late-life depression, late-onset depression, dementia, Alzheimer’s disease (AD), vascular dementia, prodromal depression, cognitive impairment, prodromal depression, and behavioral and psychological symptoms of dementia. Priority was given to longitudinal cohort studies, meta-analyses, neuroimaging and biomarker studies, and major randomized controlled trials relevant to older adults. Reference lists of key articles were additionally screened to identify influential studies not captured in the initial search.

In this review, late-life depression refers to depressive syndromes occurring in older adults, typically aged ≥60 years, regardless of age at first onset [23,24]. Late-onset depression is used more specifically to denote first-ever depressive episodes emerging in later life (commonly after age 60–65), a phenotype often associated with cerebrovascular and neurodegenerative vulnerability [2]. The term prodromal depression is used to denote depressive symptoms that occur in close temporal proximity to dementia onset and are accompanied by supportive biological evidence of underlying neurodegenerative or vascular pathology, analogous to established prodromal frameworks in other neurodegenerative disorders [25,26]. Executive dysfunction refers to impairments in cognitive control processes such as set-shifting, inhibition, and working memory, typically reflecting fronto-subcortical circuit involvement [27]. Subjective cognitive decline (SCD) denotes self-experienced worsening of cognitive function in the absence of objective impairment on standard neuropsychological testing [28].

## 3. Shared and Distinct Patterns of Cognitive Impairment in Depression and Dementia

Cognitive impairment in late-life depression and dementia frequently involves deficits in attention, processing speed, and executive function, thereby creating a significant overlap in neuropsychological profiles. For example, older adults with major depressive disorder often demonstrate reductions in processing speed and executive functioning tasks, pointing to dysfunction within frontostriatal circuits [29,30]. Similarly, in the early stages of dementia, the decline in attention and processing speed may precede overt memory impairment, implicating frontal lobe involvement in both conditions [31,32]. This overlap poses a diagnostic challenge in distinguishing a reversible “pseudo-dementia” picture from a progressive neurodegenerative process, underscoring the need for nuanced neuropsychological assessment. However, the extent and pattern of cognitive impairment in late-life depression are highly heterogeneous and vary according to depression subtype (e.g., melancholic vs. non-melancholic), symptom severity, treatment status, and cognitive reserve, which further complicates differential diagnosis in clinical practice.

Despite these shared features, important distinctions in cognitive profiles emerge between depression and dementia. Memory dysfunction in AD typically involves impaired encoding and storage of new information [33], whereas in depression the memory impairment tends to be more retrieval-based with recognition relatively spared [34]. This distinction is not absolute, as severe depression, mixed vascular pathology, or comorbid depression and dementia may show overlapping memory profiles. In addition, cognitive deficits in depression may demonstrate substantial recovery following effective mood treatment, indicating a degree of reversibility; by contrast, cognitive decline in dementia is characteristically progressive and largely irreversible [9,35]. This divergence in trajectory and reversibility strengthens the conceptual distinction between functional cognitive impairment and early neurodegeneration.

From a neurobiological and neuroimaging perspective, depression and dementia share disruption in frontal–subcortical and limbic networks, yet they diverge in anatomical and pathophysiological patterns [36,37,38]. In late-life depression, evidence points to altered dorsolateral prefrontal cortex and anterior cingulate cortex functioning, increased WMH burden, and a “vascular depression” pattern that implicates cerebrovascular burden [36,38]. By contrast, dementia—particularly AD—exhibits prominent hippocampal and medial temporal-lobe atrophy, posterior cortical hypometabolism, and accumulation of amyloid and tau pathology [39]. Thus, while both conditions may involve frontolimbic network disruption, the underlying substrate in depression tends to be more vascular/functional, whereas in dementia it is more degenerative/structural; however, substantial overlap exists, particularly in mixed pathologies and in vascular contributions to AD.

Clinically, the recognition of this continuum from depression through cognitive impairment to dementia has considerable implications. Older adults presenting with late-onset depression, marked executive dysfunction, and high WMH burden are at increased risk of subsequent cognitive decline and conversion to dementia [23,40]. Accordingly, neuropsychological assessment in depressed older patients should incorporate not only mood-symptom resolution but also longitudinal cognitive monitoring, especially in the domains of processing speed and executive control, at regular intervals (e.g., annually or with clinical change) with escalation to neuroimaging or biomarker evaluation when cognitive decline persists despite mood remission or is accompanied by prominent executive dysfunction or high WMH burden [41]. Understanding both the similarities and differences in cognitive impairment between depression and dementia enables more precise stratification of risk and informs timely intervention strategies.

## 4. Depression as a Risk Factor for Dementia

Meta-analytic evidence strongly supports depression as an independent risk factor for subsequent dementia. In this context, depression is conceptualized as a risk factor when it precedes dementia by a prolonged latency period and occurs in the absence of biological markers indicating imminent neurodegenerative change. In a comprehensive meta-analysis of 23 cohort studies, Diniz et al. (2013) reported that late-life depression was associated with an 85% increased risk of all-cause dementia (pooled odds ratio [OR] 1.85, 95% confidence interval 1.67–2.04), with subtype analyses showing a stronger association for vascular dementia (OR 2.52) than for AD (OR 1.65) [1]. Similar findings have been replicated in other large population-based cohorts and systematic reviews, consistently demonstrating that depressive disorders across the lifespan are linked to higher incidence of MCI and dementia [42,43]. More recent nationwide data from Denmark confirmed this association across early-, mid-, and late-life depression, showing HRs of 3.08, 2.95, and 2.31, respectively, after adjustment for education, income, cardiovascular disease, diabetes, and major psychiatric comorbidities [44]. The impact of depression on dementia risk also appears to vary by age: a recent Korean cohort study of adults aged ≥ 75 years suggested that the association weakens with advancing age [45]. Importantly, accumulating evidence suggests that depression severity, chronicity, and treatment resistance further modify dementia risk. Individuals with recurrent, persistent, or treatment-resistant depression appear to have a substantially higher risk of subsequent dementia than those with milder or remitting depressive episodes, even after adjustment for major confounders. A recent large-scale longitudinal study demonstrated a graded increase in dementia risk with greater cumulative depressive symptom burden and poor treatment response, highlighting depression severity as a clinically relevant risk stratifier [46].

Several biological mechanisms have been proposed to explain the link between depression and later dementia. Among these, vascular pathology appears to play a particularly prominent role. Neuroimaging and neuropathological studies have consistently shown that older adults with depression often exhibit increased WMH, lacunar infarcts, and small-vessel disease, collectively forming the substrate of the so-called “vascular depression” subtype [14,36]. These cerebrovascular alterations may disrupt frontostriatal and limbic circuits that are essential for executive and emotional regulation, providing a plausible mechanistic bridge between mood disturbance and cognitive decline [11]. Vascular pathology may act both as a shared etiological factor contributing to the emergence of depression and cognitive decline, and as a mediator through which depression accelerates neurodegenerative trajectories. Conversely, while AD pathology—amyloid-β and tau accumulation—has been postulated to underlie the depression-dementia relationship, biological evidence for a direct association remains inconsistent. A recent systematic review and meta-analysis of cerebrospinal fluid, positron emission tomography (PET), and plasma studies found no overall association between amyloid-β burden and depression in older adults without dementia, although subgroup analyses suggested a possible link in cognitively impaired individuals [47]. Subgroup signals have been reported primarily in individuals with co-occurring MCI or late-onset depression, although subgroup definitions and biomarker thresholds vary substantially across studies [25,47,48]. Emerging evidence also implicates neuroinflammatory and oxidative mechanisms in the depression–dementia continuum, with elevated peripheral inflammatory markers such as interleukin-6 being linked to late-life depression [49], microglial activation and HPA-axis dysregulation contributing to neurodegenerative vulnerability [50], and oxidative stress biomarkers such as nitrotyrosine predicting subsequent dementia in depressed older adults [51]. Thus, while depression may coexist with Alzheimer’s pathology in some individuals, current evidence indicates that alternative biological pathways, rather than amyloid–tau accumulation alone, may contribute to the observed association with cognitive decline. These pathways likely represent partially overlapping processes, with inflammatory and HPA-axis alterations primarily supported by cross-sectional associations, whereas oxidative stress markers have shown more consistent longitudinal associations with subsequent cognitive decline [49,50,51].

Interpretation of the association between depression and dementia risk requires careful consideration of confounding and causal inference. Depression and dementia share multiple upstream determinants, including vascular risk factors (e.g., hypertension, diabetes, and cerebrovascular disease), frailty, social isolation, sleep disturbance, and cognitive reserve [52,53,54,55]. Large cohort studies have attempted to address these issues through multivariable adjustment, exclusion of baseline cognitive impairment, lag-time analyses, and sensitivity analyses stratified by vascular comorbidity [44,56,57]. Nevertheless, residual confounding and reverse causality remain important methodological challenges, particularly in late-life depression where depressive symptoms may emerge in close temporal proximity to dementia onset [58,59]. These limitations underscore the need to interpret epidemiological associations with caution and to integrate longitudinal, biomarker-informed, and mechanistic evidence when inferring causal pathways.

## 5. Depression as a Prodromal Symptom of Dementia

In contrast to depression acting as a distal risk factor, prodromal depression is conceptualized as depressive symptoms that emerge in close temporal proximity to dementia onset and are accompanied by clinical or biological features suggestive of underlying neurodegenerative or vascular pathology [25,26]. Accumulating longitudinal data suggest that in a subset of older adults, depressive symptoms may function as a prodromal manifestation of an incipient neurodegenerative process. For example, a large cohort study found that individuals with late-onset depression exhibited a significantly elevated rate of dementia onset within the first few years of diagnosis, indicating temporal closeness to dementia onset rather than a lengthy latency period [60]. Moreover, systematic reviews have debated whether late-onset depression represents an early stage of AD or vascular dementia pathology rather than merely a comorbid mood disorder [58]. These observations highlight the importance of viewing certain episodes of late-life depression not just as antecedent risk factors, but as clinical signals of evolving brain pathology.

Although prodromal depression is difficult to distinguish clinically from non-prodromal forms of late-life depression, biomarker studies suggest that biological differences may emerge in specific subgroups. For example, Wu et al. (2016) [48] reported higher cortical amyloid burden in patients with late-life depression compared with healthy controls using ^18^F-florbetapir PET; however, this association was confined to individuals with co-occurring amnestic MCI and has not been uniformly replicated across studies, underscoring its subgroup-specific and emerging nature. Late-onset depression has been associated with a higher burden of WMH and fronto-subcortical circuit disruption, which correlate with greater cognitive impairment [61]. Moreover, older adults with depression who exhibit substantial WMH burden tend to show poorer antidepressant treatment response, a pattern that is consistent with the involvement of cerebrovascular pathology in both affective and cognitive outcomes rather than establishing a direct causal relationship [14,36]. In one large study, the combination of late-life depression and SCD was associated with substantially higher incidence of MCI and dementia compared with either condition alone [62]. These features suggest that depressive episodes occurring later in life may mask or overlap with very early neurodegenerative or vascular brain changes.

However, despite growing interest in prodromal depression as an early manifestation of dementia, biological evidence supporting this interpretation remains limited and heterogeneous. Neuroimaging and biomarker studies suggesting increased amyloid burden or vascular pathology in late-life depression are largely subgroup-specific, cross-sectional, and not uniformly replicated, with generally modest effect sizes. In particular, meta-analytic evidence has not demonstrated a consistent association between amyloid-β burden and depressive symptoms in cognitively unimpaired older adults, underscoring that late-life depression cannot be regarded as a definitive biological prodrome of dementia in most patients. These limitations highlight the need for cautious interpretation and for longitudinal, biomarker-informed studies to clarify temporal sequencing and causal direction.

Recognizing depression as a potential prodromal symptom of dementia has important prognostic and clinical ramifications. Older adults with new-onset depression—especially those exhibiting executive dysfunction, SCD, and neuroimaging markers of cerebrovascular or hippocampal damage—appear to progress more rapidly to dementia than those without such features [63]. Accordingly, longitudinal cognitive monitoring is particularly warranted in older adults with new-onset depression who exhibit executive dysfunction, subjective cognitive decline, or marked WMH burden, as these features may signal a higher-risk trajectory and should prompt consideration of neuroimaging, vascular risk evaluation, or biomarker referral. Early detection of the transition from depressive state to cognitive decline may facilitate timely intervention and possibly delay or mitigate progression to full-blown dementia.

## 6. Depression as a BPSD

Depressive symptoms—including clinically significant depressive syndromes rather than strictly defined major depressive disorder—are among the most common BPSD, affecting an estimated 30–50% of patients with AD and even higher proportions in dementia with Lewy bodies (DLB) and frontotemporal dementia (FTD) [17,18]. The prevalence of clinically significant depression is particularly elevated in DLB, where affective symptoms frequently coexist with fluctuations in attention and visual hallucinations [64], and in FTD—especially the behavioral variant—where apathy and mood changes are prominent early features [65]. The presence of depression in dementia is associated with faster cognitive and functional decline, greater neuropsychiatric burden, increased rates of institutionalization, and reduced quality of life for both patients and caregivers [66,67]. Several longitudinal studies further indicate that dementia patients with comorbid depression experience higher mortality and are more likely to exhibit severe agitation, psychosis, and loss of independence, even after adjustment for baseline dementia severity, vascular comorbidity, and neuropsychiatric symptom burden [68]. In one longitudinal cohort of patients with moderate-stage, mixed-etiology dementia (baseline Mini-Mental State Examination [MMSE] ≈ 19), multilevel mixed-effects modeling estimated an average decline of 13.7 MMSE points over 3 years, with comorbid major depression associated with an additional model-based loss beyond the baseline trajectory [69]. These epidemiological and clinical findings underscore that depression emerging in the context of established dementia is not a benign comorbidity but a major determinant of disease trajectory and patient outcomes.

Depressive symptoms in dementia substantially overlap with apathy, yet these syndromes are clinically and prognostically distinct [18,68]. While depression is characterized by subjective distress, sadness, guilt, and negative affect, apathy primarily reflects diminished motivation, emotional blunting, and reduced goal-directed behavior, often in the absence of overt dysphoria [18,21,65]. In dementia syndromes—particularly AD, DLB, and FTD—apathy is more prevalent than major depressive episodes and is more strongly associated with accelerated cognitive decline, functional deterioration, and caregiver burden [21,66,69]. Antidepressants may alleviate depressive affect but show limited efficacy for apathy [70,71], whereas cholinesterase inhibitors and non-pharmacological interventions targeting engagement and environmental stimulation appear more beneficial for apathy-related symptoms [72,73,74]. Failure to differentiate depression from apathy may therefore obscure treatment response and prognostic assessment in patients with dementia.

In established dementia, depressive symptoms have been linked to several neurobiological mechanisms supported by varying levels of evidence. Neuroimaging studies consistently demonstrate structural atrophy in limbic and paralimbic regions, including the anterior cingulate cortex, hippocampus, and orbitofrontal cortex [37], as well as disruption of default-mode and salience networks [21]. Evidence for monoaminergic degeneration involving serotonergic and noradrenergic nuclei is largely indirect, derived from neuropathological and pharmacologic observations [75]. Neuroinflammatory activation and cholinergic deficits have been proposed as contributory mechanisms, primarily based on biomarker and associative studies, particularly in AD and DLB [76,77]. Treatment response is also distinct from depression in non-demented individuals. Large randomized trials have shown limited or modest efficacy of selective serotonin reuptake inhibitors (SSRIs) in AD [70], and symptom improvement is often heterogeneous and incomplete [78]. Given this limited and inconsistent benefit, antidepressants should be prescribed cautiously in dementia, with careful consideration of overall clinical context and potential risks. In contrast, cholinesterase inhibitors and multimodal non-pharmacological interventions, including caregiver training and structured activity programs, have been shown to confer modest benefits for depressive and apathy-related symptoms [72,79]. These mechanistic and therapeutic patterns highlight the need for dementia-specific approaches when managing depression as part of the broader BPSD syndrome. Notably, support for these mechanisms varies by evidence type, with monoaminergic and limbic changes primarily supported by neuropathological and imaging studies, and inflammatory and cholinergic contributions inferred largely from biomarker and clinical association studies.

Beyond efficacy, pharmacologic management of depression and BPSD in dementia requires careful consideration of geriatric safety and prescribing trade-offs. Antidepressants, particularly SSRIs, are associated with increased risks of falls, hyponatremia, QT prolongation, bleeding, and delirium in older adults with dementia, which may outweigh modest mood benefits in some patients [70,71,80]. Cognitive enhancers also carry clinically relevant safety considerations: cholinesterase inhibitors are linked to bradycardia, syncope, weight loss, and gastrointestinal adverse effects, whereas memantine may cause dizziness, confusion, and sedation, particularly in frail individuals or those with polypharmacy [73,74,81]. These risks highlight the importance of individualized, stage-specific treatment decisions that balance symptom control against safety, functional outcomes, and caregiver burden. The distinguishing clinical and biological characteristics of late-life depression, prodromal depression, and BPSD-related depression are summarized in Table 1.

## 7. Reciprocal Effects of Antidepressant and Anti-Dementia Treatments on Cognition and Mood

Antidepressant treatment in late-life depression has been associated not only with mood improvement but also with subtle cognitive benefits in specific domains. Several clinical studies have shown that SSRIs may enhance processing speed, executive functioning—most consistently detected on tests such as the Digit Symbol Substitution Test and Trail Making Test—in older adults without dementia, particularly when depressive symptoms remit adequately [82,83]. These effects are generally modest and often statistically detectable rather than clearly clinically transformative. Agents with multimodal serotonergic actions, such as vortioxetine, have demonstrated pro-cognitive effects primarily in executive function and processing speed that appear partly independent of mood improvement in non-demented older adults, although effect sizes remain small [84,85]. In contrast, randomized trials in AD have produced inconsistent findings. The HTA-SADD trial and other large controlled studies found no significant advantage of SSRIs over placebo for depressive symptoms or cognitive outcomes in AD, underscoring the limited cognitive efficacy of conventional antidepressants once neurodegeneration is established—likely reflecting trial-design constraints such as advanced disease stage with ceiling effects, diagnostic heterogeneity, limited sensitivity of cognitive endpoints, and substantial placebo response [70,71,80]. Thus, while mood treatment may produce modest cognitive gains in non-demented older adults, such effects diminish substantially in the context of progressive dementia.

Conversely, anti-dementia pharmacotherapies appear to exert modest benefits on depressive and apathy-related symptoms, largely reflecting reductions in overall neuropsychiatric burden rather than direct antidepressant effects. Cholinesterase inhibitors, including donepezil, rivastigmine, and galantamine, have been reported to reduce neuropsychiatric symptoms in AD and related disorders, with small but measurable improvements observed across several NPI domains, including in depression, irritability, and apathy [73,74]. These effects are more consistently observed in DLB, where enhancement of cholinergic transmission may alleviate mood and behavioral symptoms alongside improvements in attention and cognitive fluctuations. Rivastigmine, in particular, has shown benefit in DLB, where enhancement of cholinergic transmission may alleviate mood symptoms alongside improvements in attention and behavioral fluctuations [86]. Memantine, an N-methyl-D-aspartate receptor antagonist, has also been associated with reductions in agitation and irritability, with inconsistent and generally modest effects on depressive symptoms, suggesting indirect mood benefits secondary to behavioral stabilization rather than primary antidepressant action [81]. Overall, although the magnitude of these effects is modest, the evidence suggests that targeting cholinergic and glutamatergic dysfunction in dementia may have secondary benefits on mood and behavioral outcomes.

Emerging disease-modifying treatments further highlight the complex interplay between cognition and affect in neurodegenerative disorders. Anti-amyloid monoclonal antibodies such as lecanemab [87] and donanemab [88] have demonstrated clinically meaningful slowing of cognitive decline in early AD; however, current trial data do not provide clear evidence of improvement in depressive symptoms, and any potential mood effects remain speculative [22,89]. Similar uncertainty surrounds tau-targeting therapies, which are primarily designed to modify neurodegenerative progression rather than affective symptoms [90]. Nevertheless, mood outcomes have not been primary endpoints in disease-modifying trials, and available data are insufficient to determine whether reductions in pathological burden translate into clinically meaningful improvements in depressive symptoms [22]. Among non-pharmacological approaches, caregiver-focused and activity-based interventions (e.g., structured exercise and engagement programs) have the most consistent evidence for improving mood and overall neuropsychiatric burden in dementia populations. Cognitive rehabilitation and neuromodulation techniques, such as repetitive transcranial magnetic stimulation, show emerging but less consistent evidence, primarily in late-life depression or early-stage cognitive impairment rather than established dementia [91,92,93]. Interventions that improve both mood and cognition may also confer downstream benefits in functional and motor domains, including gait confidence, mobility, and activities of daily living, thereby enhancing overall clinical relevance in older adults. Together, these findings point to an emerging therapeutic paradigm in which interventions increasingly target the intertwined trajectories of cognitive decline and affective disturbance in late life. Table 2 provides a narrative overview of the direction and strength of evidence across treatment classes, distinguishing evidence type and primary outcome domains rather than implying comparative efficacy.

## 8. Conclusions

Depression and cognitive impairment in late life are deeply interconnected. They share overlapping clinical manifestations and partially convergent neurobiological pathways. Evidence across epidemiological, neuroimaging, and biomarker studies indicates that depression can function both as an independent risk factor for dementia and, in selected individuals, as a prodromal expression of emerging neurodegenerative or vascular pathology. Depression may function as a risk factor when it precedes cognitive decline by a prolonged latency period, whereas a prodromal role is suggested in the context of late-onset depression occurring close to dementia onset, particularly when accompanied by executive dysfunction, WMH burden, and other markers of cerebrovascular pathology, for which evidence is more consistent than for amyloid-related mechanisms in cognitively unimpaired samples. At the same time, a substantial proportion of patients with established dementia experience depressive symptoms as part of the broader BPSD spectrum, where mood changes are associated with accelerated functional decline, greater neuropsychiatric burden, and poorer quality of life for both patients and caregivers. These stage-dependent relationships underscore the importance of moving beyond a binary distinction between “depression” and “dementia”, and instead adopting an integrative conceptual framework that captures their dynamic and bidirectional interactions. Clinically, this framework supports a staged approach in which late-onset depression with cognitive or vascular markers prompts longitudinal monitoring, targeted risk stratification, and timely escalation to multimodal intervention.

Therapeutically, the reciprocal effects of mood- and cognition-targeted treatments highlight both opportunities and limitations. Antidepressants may yield modest benefits in executive function and processing speed in non-demented older adults, but their efficacy is substantially reduced in established dementia. In dementia, cholinesterase inhibitors, memantine, and multimodal non-pharmacological interventions primarily reduce apathy and broader neuropsychiatric symptoms, with less consistent effects on depressive mood per se. Meanwhile, emerging disease-modifying therapies may alter the trajectory of Alzheimer’s pathology; however, mood outcomes have not been primary trial endpoints, and current evidence does not support a direct antidepressant effect. These findings emphasize the need for personalized, stage-specific assessment and management strategies, incorporating longitudinal cognitive monitoring, vascular and neuropsychiatric risk evaluation, and multimodal interventions tailored to the evolving clinical profiles of aging individuals, with escalation to neuroimaging or biomarker assessment warranted in the presence of persistent executive dysfunction, worsening memory profiles, high WMH burden, or rapid functional decline. The interplay between mood and cognition in late life has direct implications for real-world function, including mobility, gait stability, fall risk, and independence in instrumental activities of daily living.

## Figures and Tables

**Table 1:** Comparative Clinical Features Across Depression Types.

Feature	Depression in Non-Demented Older Adults	Depression as a Prodrome of Dementia	Depression as a BPSD
Typical onset	Any age, recurrent [11,43]	Late-onset (>60–65 yr), new-onset [25,43]	After dementia onset [16,17,18]
Core symptoms	Mood, anhedonia, psychomotor changes [7,11]	Mood, executive dysfunction, SCD or amnestic MCI [25,27,28]	Mood, apathy, emotional lability [18,21,65]
Cognitive profile	Processing speed ↓, EF ↓; memory relatively intact [7,23,29]	Processing speed ↓↓, EF ↓↓; early memory issues [25,28,48]	Global decline, attention fluctuation [17,64,65]
Neurobiology	HPA dysregulation, inflammation [11,49,50]	WMH ↑, fronto-subcortical disruption, early AD markers [14,36,48]	Monoaminergic degeneration, limbic atrophy, cholinergic deficits [37,75,77]
Risk of dementia	Increased [1,42,43]	High short-term conversion risk in enriched subgroups [28,44]	Accelerates decline [21,66,69]
Treatment response	Generally good [19,20,82]	Often poor [24,27,36]	Poor to modest [70,78,80]
Evidence level	Multiple longitudinal cohorts/meta-analyses [1,42,43]	Limited replication; subgroup biomarker evidence [25,28,48]	Multiple longitudinal co-horts/meta-analyses [16,70,80]

Abbreviations: EF, executive function; HPA, hypothalamic–pituitary–adrenal; SCD, subjective cognitive decline; MCI, mild cognitive impairment; WMH, white matter hyperintensity; BPSD, behavioral and psychological symptoms of dementia. Arrows indicate the direction and relative magnitude of change (↑ increase; ↓ decrease; ↓↓ greater decrease). Table content is based on a narrative synthesis of evidence presented in Section 3, Section 4 and Section 5.

**Table 2:** Effects of Antidepressants, Cognitive Enhancers, and Disease-Modifying Therapies on Mood and Cognition.

Treatment Class	Cognitive Effects	Mood Effects	Evidence Summary
SSRIs	Small improvements in EF/processing speed in non-dementia [19,20,83]	Mood improvement in LLD; limited in AD [20,70,80]	Strong evidence from multiple RCTs and meta-analyses in LLD [19,20,83]; limited evidence in dementia [70,71,80]
Multimodal antidepressants (Vortioxetine)	Pro-cognitive effects independent of mood [84,85]	Mood improvement [84,85]	Moderate evidence based on several RCTs and meta-analyses in non-demented older adults [84,85]
Cholinesterase inhibitors	Small improvement in attention/behavior [73,74]	Small improvement in depression/apathy [73,74,86]	Strong evidence from randomized controlled trials and meta-analyses in AD/DLB [73,74,86]
Memantine	Stabilizes cognition [81]	Reductions in agitation/irritability; mixed for depression [74,81]	Mixed evidence with heterogeneous results across trials [74,81]
Anti-amyloid antibodies (Lecanemab, donanemab)	Slows decline [87,88]	No proven effect [22,89]	Strong evidence for cognitive outcomes from phase 3 trials [87,88]; insufficient evidence for mood outcomes [22,89]
Tau therapies	Under investigation [90]	No evidence [90]	Insufficient evidence, limited to early-phase or ongoing clinical trials [90]
Non-pharm (exercise, CRT, rTMS)	Small–moderate effects [72,91,93]	Small–moderate effects [72,92,93]	Moderate and emerging evidence from heterogeneous randomized trials and systematic reviews [72,91,92,93]

Abbreviations: SSRI, selective serotonin reuptake inhibitor; CRT, cognitive rehabilitation therapy; rTMS, Repetitive transcranial magnetic stimulation; EF, executive function; LLD, late-life depression; AD, Alzheimer’s disease; DLB, dementia with Lewy bodies.

## Data Availability

Not applicable. No new data were created or analyzed in this study.

## Abbreviations

The following abbreviations are used in this manuscript:

MCI	Mild cognitive impairment
HPA	Hypothalamic–pituitary–adrenal
WMH	White matter hyperintensity
BPSD	Behavioral and psychological symptoms of dementia
AD	Alzheimer’s disease
HR	Hazard ratio
OR	Odds ratio
PET	Positron emission tomography
SCD	Subjective cognitive decline
DLB	Dementia with Lewy bodies
FTD	Frontotemporal dementia
SSRI	Selective serotonin reuptake inhibitor
